# A retrospective analysis of factors influencing re-operation in patients undergoing mechanical valve replacement

**DOI:** 10.5830/CVJA-2013-044

**Published:** 2013-10

**Authors:** Ebuzer Aydin, Fikri Yapici

**Affiliations:** Department of Cardiovascular Surgery, Dr Siyami Ersek Thoracic and Cardiovascular Surgery Training and Research Hospital, Istanbul, Turkey; Department of Cardiovascular Surgery, Dr Siyami Ersek Thoracic and Cardiovascular Surgery Training and Research Hospital, Istanbul, Turkey

**Keywords:** platelet dysfunction, re-operation, heart valve prosthesis implantation, risk factors, mortality, retrospective studies

## Abstract

**Background:**

We aimed to determine the possible factors leading to re-operation in patients undergoing mechanical valve replacement and to investigate the relationship between valvular thrombus formation and mean platelet volume.

**Methods:**

The medical records of 43 patients with mechanical valve implantation, who were admitted to the Department of Cardiovascular Surgery of Dr Siyami Ersek Thoracic and Cardiovascular Surgery Training and Research Hospital between 2000 and 2005 were analysed retrospectively. Data recorded included demographic characteristics, valve type, size and location, implantation position, warfarin use, INR level, additional cardiac intervention, presence of left atrial thrombus, valvular thrombus, pannus formation, perivalvular leak, left atrial aneurysm, platelet count and mean platelet volume (MPV), bleeding after the primary surgery and/or revision of surgery due to other reasons, valve protection, aortic root expansion, presence of valve calcification and infective endocarditis, pre- and postoperative rhythm pattern, brand name of prosthesis, distance of the patient’s house from a cardiac surgery centre, and concomitant non-cardiac systemic diseases.

**Results:**

Mean age was 49.3 years (range 19–78 years). Of the patients, 51% (*n* = 22) were males and 49% (*n* = 21) were females. The re-operation mortality was 11.6%. Age, gender, valve type, brand of valve prosthesis, and implantation position were not risk factors for re-operation. The MPV was higher and statistically significant in patients with valvular thrombus during re-operation (*p* < 0.001). MPV was determined to be an independent risk factor with 85% sensitivity and 87% specificity.

**Conclusion:**

MPV and INR levels should be closely monitored when designing individualised postoperative medical treatment for patients undergoing heart valve re-operation.

## Abstract

Currently, cardiac valve diseases that require surgery are mainly due to stenosis, insufficiency, and fixed valves in stenosis accompanied by insufficiency. Acute rheumatic fever is the main cause of mitral insufficiency in developing populations.^1^ Improved survival rate after the primary surgery has led to increased numbers of re-operations.^2^ Therefore, in recent years there has been a tendency towards increasing age of patients undergoing re-operations on heart valve prostheses.^3^

The major causes of re-operation include progression of postoperative native valve disease after non-valve surgery, and structural degeneration of bioprosthetic surgical valves. Re-operations are more complicated than the initial procedure due to adhesive processes around the heart, and the common association of pulmonary hypertension (PHT). In addition, replacement operations are often performed in functionally compromised patients who tolerate complications less well.^4^ In the past, re-operative valve surgery was associated with a higher mortality rate than with primary valve operations; however, mortality and morbidity rates have decreased recently.^2^,^4^,^5^

Although mechanical valves are long-lasting in young patients, there is an increased risk for thrombogenic, particularly thromboembolic, events and this requires longterm anticoagulation therapy. Such thromboembolic events are dependent on valve design, materials used and characteristics of the patient.^6^ Endocarditis, dehiscence, perivalvular leak and pannus formation are commonly encountered with mechanical and biological valves, while acute prosthetic valve thrombosis is the main complication of mechanical valves.

In this study, we aimed to determine possible factors leading to re-operation in patients undergoing mechanical valve replacement and also to investigate the relationship between valvular thrombus formation and mean platelet volume (MPV).

## Methods

Between 2000 and 2005, 2 141 patients underwent heart valve surgery in the Department of Cardiovascular Surgery of Dr Siyami Ersek Thoracic and Cardiovascular Surgery Training and Research Hospital, Istanbul, Turkey. Of the patients, 1 615 had valve surgery, 370 had valve surgery with coronary artery bypass graft (CABG) surgery, and 156 had mitral valve repair. During the same time interval, the number of re-operations was 176.

Retrospective analysis was performed on the medical data of 60 re-operated patients, but 17 were excluded from the study due to missing data. Therefore, the study group was made up of 43 patients who had undergone mechanical valve implantation. Patients who underwent bioprosthetic valve replacement during the primary surgery were also excluded from the study to achieve more homogenous results and to investigate whether each parameter, namely valvular thrombus formation, pannus formation and perivalvular leak, was an independent risk factor for valve dysfunction and re-operation.

Data recorded included age, gender, valve type, valve size, valve location, implantation position, warfarin use, INR level, additional cardiac intervention, presence of left atrial thrombus, valvular thrombus, pannus formation, perivalvular leak, left atrial aneurysm, platelet count and MPV, bleeding after the primary surgery and/or revision surgery due to other reasons, valve protection, aortic root expansion, presence of valve calcification and infective endocarditis, pre-operative and postoperative rhythm pattern, brand name of prosthesis, distance of the patient’s house from a cardiac surgery centre, and concomitant non-cardiac systemic diseases. The factors potentially leading to re-operation included valvular thrombus formation, pannus formation and perivalvular leak.

The study protocol was approved by the local ethics committee.

## Statistical analysis

This was performed using Windows SPSS v13.0 software. Data were expressed as percentage. A *p*-value of < 0.05 was considered significant. Categorical data were expressed as number and percentage, while numerical data were expressed as mean ± standard deviation. Pearson’s chi-square test and Fisher’s exact test were used for non-parametric variables, while the Mann-Whitney *U*-test was performed for parametric variables. A linear regression analysis was also performed for significant parameters. A ROC curve was drawn to determine the sensitivity and specificity of these parameters.

## Results

The mean age of patients was 49.3 years (range 19–78 years); 51% (*n* = 22) were males and 49% (*n* = 21) were females. Twenty-nine patients underwent mitral valve replacement, while 12 underwent aortic valve replacement. A tricuspid valve was implanted in two patients.

There was no statistically significant difference in baseline demographic characteristics of the patients. Age and gender were not a determining factor for re-operation. Demographic characteristics of the patients and distribution of indications for re-operation are summarised in Table 1 and 2, respectively.

**Table 1:** Demographic Characteristics Of The Patients

*Variables*	*Number (n)*	*Percentage (%)*
Gender		
Male	22	51
Female	21	49
Valve replacement		
Mitral	29	67.4
Aortic	12	27.9
Tricuspid	2	4.6
Implantation position		
Anatomical	33	76.7
Extra-anatomical	10	23.2
Additional cardiac intervention	10	23.2
Concomitant non-cardiac disease	3	6.9
Concomitant cardiac disease	3	6.9
Infective endocarditis	13	30.2
Perivalvular leak	23	53.4
Valve calcification	17	39.5
Pannus formation	19	44.1

**Table 2:** Distribution Of Indications For Re-Operation Among Patients

Indications*	*Number of patients*
Perivalvular leakage	23
Thrombus formation	21
Pannus formation	19
Valvular calcification	17
Infective endocarditis	13
Additional cardiac intervention	10
Left atrial thrombus	6
Accompanying cardiac disease	3
Left atrial aneurism	2

*There were patients with more than one indication for re-operation.

The incidence of thrombus formation in mechanical prosthetic valves was statistically significantly higher in patients with valve calcification (*p* < 0.05) and left atrial thrombus (*p* = 0.007) during the primary surgery. Pearson’s chi-square test revealed that the incidence of perivalvular leak was higher in patients with left atrial thrombus during the primary surgery (*p* < 0.05). The incidence of perivalvular leak was statistically higher in patients with valvular thrombus and pannus formation (*p* < 0.05). In addition, the incidence of perivalvular leak was higher in patients with infective endocarditis compared with those without the disease (*p* < 0.05).

**Fig. 1.**
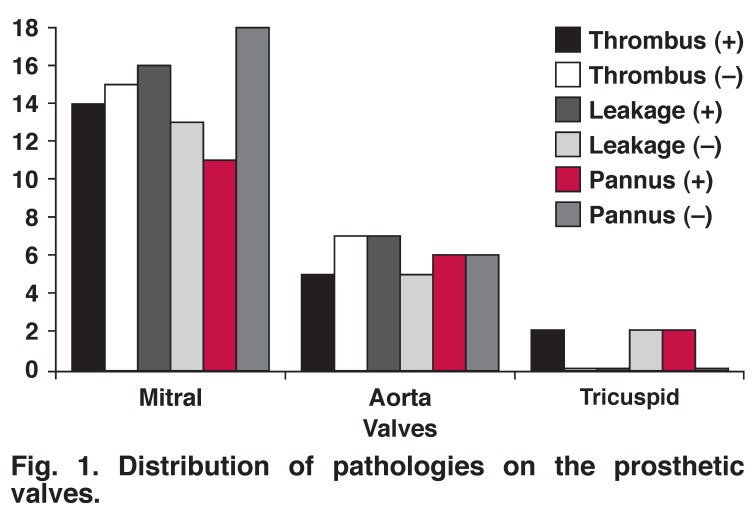
Distribution of pathologies on the prosthetic valves.

The re-operation mortality rate was 11.6%. A total of 67.4% (*n* = 29) of patients had mitral valve disease, while 27.9% (*n* = 12) had aortic valve disease. A mitral valve was implanted anatomically and extra-anatomically in 22 and seven patients, respectively. It was observed that valve type and implantation position were not risk factors for re-operation. A St Jude (St Jude Medical Inc, Minnesota, USA) prosthetic valve was implanted in 81.4% of patients, a Carbomedics valve (SuzerCarbomedics Inc, Austin, Texas, USA) was implanted in 7% of patients, and a Medtronic valve (Medtronic Inc, Minnesota, USA) was implanted in 7% of patients. The brand of valve prosthesis was not a risk factor for re-operation (Fig. 1).

**Fig. 2.**
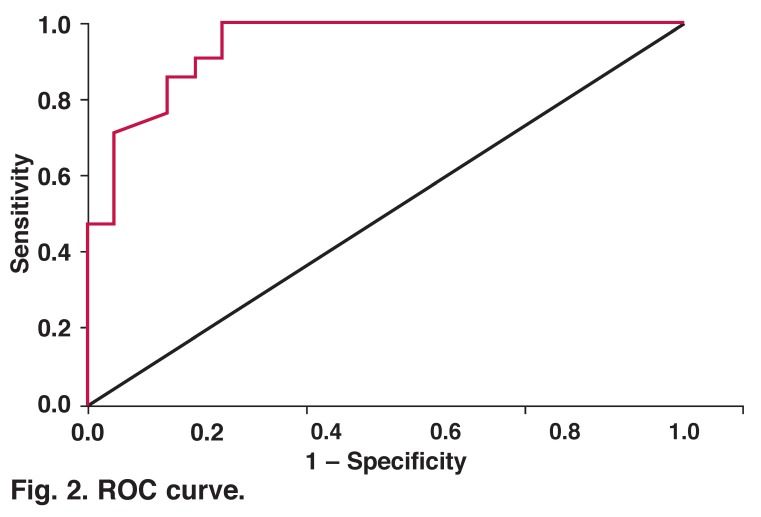
ROC curve.

The mean platelet volume was higher and statistically significant in patients with valvular thrombus during re-operation (*p* < 0.001). A linear regression analysis was performed of parameters that were statistically significantly related to valvular thrombus, including left atrial thrombus, MPV, valve calcification, and perivalvular leak. It was observed that there was a statistically significant impact of these four parameters on valvular thrombus formation (*R* = 0.60). However, MPV was an independent risk factor (*p* < 0.001). A ROC curve showed a higher percentage of sensitivity (85%) and specificity (87%) (Fig. 2).

## Discussion

Although surgical modalities and myocardial protection techniques have been improved recently, the mortality rate of heart valve re-operation varies between 10 and 20%.^7^ This leads to increased cost of care and work load of surgical centres. Delay in re-operation also results in increased morbidity and mortality, particularly in developing countries. Such undesired outcomes may be prevented by defining the factors that lead to re-operation and designing a preventive healthcare policy.

Overall complications observed with prosthetic heart valves are divided into six main categories: structural valvular deterioration, non-structural dysfunction, valve thrombosis, embolism, bleeding and endocarditis. While leaflet calcification and leaflet tearing are more commonly encountered with bioprosthetic heart valve implantation, haemolysis, platelet activation and thromboembolic events resulting from clot formation are commonly encountered in mechanical heart valves.^8^

Biomedical engineering studies revealed that these complications might be related to non-physiological blood flow patterns in the vicinity of the heart valves. In fact, the potential of abnormal flow patterns to promote blood cell damage has long been recognised. Abnormal flow patterns cause thrombus formation by imposing forces on cell elements in high shear-stress regions (so leading to haemolysis and platelet activation), and changing the frequency of contact (particularly activating platelets for thrombus formation). In addition, these abnormal flow patterns might induce leaflet calcification, and tearing in tissue and polymeric valves by high shear-force regions near the leaflet surfaces.^8^,^9^

For clinical interpretation, it has been reported in the literature that mechanical valve dehiscence and pannus formation were encountered with both bioprosthetic and mechanical valves. Acute prosthetic valve thrombosis was commonly seen with mechanical valves only.^10^

In line with the literature, our results demonstrated that thrombus and pannus formation were the leading indications for re-operation, whereas left atrial thrombus and valvular calcification were diagnosed in nearly one-quarter of the sample group. We believe that a high risk of thrombus formation in patients with left atrial thrombus and valvular calcification in the initial surgery might indicate a tendency to thrombosis and ongoing inflammatory processes. Correlated with this process, MPV may indicate increased risk of thrombosis and the inflammatory process.

No or inadequate oral anticoagulation (INR < 2.5) or extreme fluctuations of INR values were also reported as strong risk factors in the aetiology of mechanical prosthetic valve thrombosis.11 In our study, no direct correlation was determined between valvular thrombus formation and anticoagulant use. Similarly, we did not observe any statistically significant relationship between warfarin use/INR levels and thrombosis (*p* > 0.05).

The distance of the patient from a cardiac surgery centre was another major factor in the management of the condition. The postoperative life expectancy of patients undergoing prosthetic valve replacement was closely related to reversibility of myocardial or other organ damage. Therefore, the timing of the operation was critical.^6^ Bioprosthetic valves may be more useful for patients who live in rural areas.

Although there was a statistically significant relationship between the presence of calcification in the native valve and valvular thrombus during the primary surgery (*p* > 0.05), it was not significantly related to perivalvular leak (*p* > 0.5). However, the incidence of perivalvular leak was expected to be increased in patients with annular calcification. This may have been caused by the small sample size of the study.

The aortic annulus is not only a circular structure that supports the leaflets of the heart valve, it also allows opening of the leaflets during the ventricular systole. The anatomical structure and shape of the aorta as well as the ventricular outflow tract are of utmost importance for maintaining blood flow. Considering these anatomical and physiological characteristics, non-conformity of the prosthetic valve and annulus may lead to perivalvular leak.

In a study by Beghi *et al*., it was shown that perivalvular leak led to aortic valve replacement.^12^ The factors leading to perivalvular leak include prosthetic valve endocarditis, Marfan syndrome, bicuspid aorta and a highly calcific aortic annulus. The major site for perivalvular leak is the mid-zone of the non-coronary sinus and the right coronary sinus.^13^ In the present study, we suggest that the anatomical structure of the zone resulted in perivalvular leak. In addition, Thubrikar *et al*. demonstrated that non-conformity of the prosthetic valve and aortic annulus led to perivalvular leak in these zones.^14^

In another study investigating reasons for prosthetic mitral valve regurgitation, it was shown that 46% of the patients underwent re-operation due to structural degeneration of the valve.^15^ Structural degeneration was defined as valve stenosis or regurgitation secondary to calcification or ruptured leaflets, whereas non-structural degeneration was defined as valve stenosis or regurgitation secondary to trauma, pannus formation or surgery. In these patients, perivalvular leak was the leading cause of re-operation in 20%, non-structural degeneration in 10%, progression of primary valve disease in 8%, and infective endocarditis in 6%.

Mitral valve re-operation did not increase the length of hospital stay, compared to the primary surgery. In addition, re-operation was shown not to be a risk factor for peri-operative mortality. Despite a higher incidence of perivalvular leak with mechanical valves, structural degeneration was more often seen with bioprosthetic valves. There was no difference in postoperative complications and mortality rate between the groups.

The incidence of infective endocarditis was similar between the groups. A total of 30.2% of the patients had infective endocarditis in our study. We also defined a statistically significant relationship between infective endocarditis and perivalvular leak (*p* = 0.043). As anticipated, infection resulted in structural degeneration and eventually perivalvular leak. Infective endocarditis also increased the risk for valvular thrombus. This may have been due to the low sociocultural status of the patients, and impaired communication between the patient and treating physician to provide effective and suitable medical treatment.

There are studies in the literature investigating the role of MPV in various conditions, such as coronary artery ectasia, outcome after myocardial infarction, unstable angina, coronary artery disease in haemodialysis patients, atherosclerosis and coronary heart diseases.^16^-^21^ MPV was reported to be an indicator of platelet activation, which resulted in development of thrombosis. It was found that large platelets contained denser, more active granules metabolically and enzymatically, with higher thrombotic potential. Therefore, they secreted more pro-thrombotic substances, pro-coagulatory surface proteins, thromboxane A2, serotonin and β-thromboglobulin.

Taşoğlu *et al.*^22^ conducted a study on the predictive marker value of MPV in patients with mechanical valve thrombosis. They reported that MPV may be used effectively during follow up of these patients. Similarly, we determined that MPV values were higher in patients with valvular thrombus, compared to the controls. This indicated a statistically significant trend. Based on the ROC curve, MPV had more than 80% sensitivity and specificity. However, the small sample size, non-standardised MPV values before re-operation, absence of detailed genetic examination, and thrombogenic factors were critical limitations of our study.

Many studies have been conducted using cheap but effective markers to identify the relationship between thrombosis and inflammation. As MPV is believed by some researchers to be a good candidate, it is being investigated in various cardiac diseases. MPV is therefore considered an acute-phase reactant and in recent years has been shown to be raised in inflammatory processes.^23^ Bansal *et al.*^24^ reported that the incidence of pulmonary embolism and PHT were higher in patients with chronic obstructive pulmonary disease and a higher level of MPV.

## Conclusion

Morbidity and mortality rates in heart valve re-operations can be decreased by the effective use of warfarin, the adoption of minimally invasive surgery, and the use of improved materials and advanced technology in artificial heart valve production. Moreover, MPV is an independent risk factor for valvular thrombus formation. Therefore, MPV values should be assessed and INR levels should be monitored more often when designing individualised postoperative medical treatment for patients undergoing heart valve re-operation.

## References

[1] Iung B, Vahanian A (2003). Mitral stenosis.. Ann Cardiol Angeiol.

[2] Weerasinghe A, Edwards MB, Taylor KM (1999). First redo heart valve replacement: a 10-year analysis.. Circulation.

[3] Fremes SE, Goldman BS, Ivanov J, Weisel RD, David TE, Salerno T (1989). Valvular surgery in the elderly.. Circulation.

[4] Cohn LH, Aranki SF, Rizzo RJ, Adams DH, Coqswell KA, Kinchla NM (1993). Decrease in operative risk of reoperative valve surgery.. Ann Thorac Surg.

[5] Jones JM, O’kane H, Gladstone DJ, Sarsam MA, Campalani G, MacGowan SW (2001). Repeat heart valve surgery: risk factors for operative mortality.. J Thorac Cardiovasc Surg.

[6] Rizzoli G, Guglielmi C, Toscano G, Pistorio V, Vendramin I, Bottio T (1999). Reoperations for acute prosthetic thrombosis and pannus: an assessment of rates, relationship and risk.. Eur J Cardiothorac Surg.

[7] Toker ME, Eren E, Guler M, Kirali K, Yanartas M, Balkanay M (2009). Second and third cardiac valve reoperations: factors influencing death and long-term survival.. Tex Heart Inst J.

[8] Dasi LP, Simon HA, Sucosky P, Yoganathan AP (2009). Fluid mechanics of artificial heart valves.. Clin Exp Pharmacol Physiol.

[9] Nobili M, Sheriff J, Morbiducci U, Redaelli A, Bluestein D (2008). Platelet activation due to hemodynamic shear stresses: damage accumulation model and comparison to in vitro measurements.. ASAIO J.

[10] Sivasubramanian S, Vijayshankar CS, Krishnamurthy SM, Santhosham R, Dwaraknath V, Rajaram S (1996). Surgical management of prosthetic valve obstruction with the Sorin tilting disk prosthesis.. J Heart Valve Dis.

[11] Bollag L, Attenhofer Jost CH, Vogt PR, Linka AZ, Rickli H, Oechslin E (2001). Symptomatic mechanical heart valve thrombosis: high morbidity and mortality despite successful treatment options.. Swiss Med Wkly.

[12] Beghi C, De Cicco G, Nicolini F, Ballore L, Reverberi C, Gherli T (2002). Cardiac valve reoperations: analysis of operative risk factors in 154 patients.. J Heart Valve Dis.

[13] De Cicco. F, Lorusso R, Colli A, Nicolini F, Fragnito C, Grimaldi T, Borrello B (2005). Aortic valve periprosthetic leakage: anatomic observations and surgical results.. Ann Thorac Surg.

[14] Thubrikar M, Nolan SP, Bosher LP, Deck JD (1980). The cyclic changes and structure of the base of the aortic valve.. Am Heart J.

[15] Potter DD, Sundt TM, Zehr KJ, Dearani JA, Daly RC, Mullany CJ (2004). Risk of repeat mitral valve replacement for failed mitral valve prostheses.. Ann Thorac Surg.

[16] Sen N, Tavil Y, Yazici HU, Hizal F, Açikgöz SK, Abaci A (2007). Mean platelet volume in patients with coronary artery ectasia.. Med Sci Monit.

[17] Martin JF, Bath PM, Burr ML (1991). Influence of platelet size on outcome after myocardial infarction.. Lancet.

[18] Pizzulli L, Yang A, Martin JF, Lüderitz B (1998). Changes in platelet size and count in unstable angina compared to stable angina or non-cardiac chest pain.. Eur Heart J.

[19] Henning BF, Zidek W, Linder B, Tepel M (2002). Mean platelet volume and coronary heart disease in hemodialysis patients.. Kidney Blood Press Res.

[20] Kario K, Matsuo T, Nakao K (1992). Cigarette smoking increases the mean platelet volume in elderly patients with risk factors for atherosclerosis.. Clin Lab Haematol.

[21] Trowbridge EA, Martin JF (1987). The platelet volume distribution: a signature of the prethrombotic state in coronary heart disease?. Thromb Haemost.

[22] Taşoğlu I, Yalçınkaya A, Ulaş MM, Lafçı G, Çiçek OF, Ulus AT (2011). Could mean platelet volume be a predictive marker for mechanical valve thrombosis?. Turkish J Thorac Cardiovasc Surg.

[23] Gasparyan AY, Ayvazyan L, Mikhailidis DP, Kitas GD (2011). Mean platelet volume: a link between thrombosis and inflammation?. Curr Pharm Des.

[24] Bansal R, Gupta HL, Goel A, Yadav M (2002). Association of increased platelet volume in patients of chronic obstructive pulmonary disease: Clinical implications. J Ind Acad Clin Med.

